# Predictive value of 1-hour postprandial SPARC levels on metabolic outcomes from Mediterranean diet adherence: results from a randomized controlled feeding study

**DOI:** 10.1093/lifemeta/loaf039

**Published:** 2025-11-13

**Authors:** Yanru Chen, Mengshan Ni, Yufei Chen, Chongrong Shen, Yaogan Luo, Huibin Lin, Juan Zhang, Huajie Dai, Aibo Gao, Muye Tong, Yinmeng Zhu, Yan Lu, Jie Hong, Weiqiong Gu, Rong Zeng, Weiqing Wang, Xu Lin, Min Xu, Ruixin Liu, Guang Ning, Jiqiu Wang

**Affiliations:** Department of Endocrine and Metabolic Diseases, Shanghai Institute of Endocrine and Metabolic Diseases, Ruijin Hospital, Shanghai Jiao Tong University School of Medicine, Shanghai 200025, China; Shanghai National Clinical Research Center for Metabolic Diseases, Key Laboratory for Endocrine and Metabolic Diseases of the National Health Commission of the PR China, Shanghai National Center for Translational Medicine, Shanghai 200025, China; Department of Endocrine and Metabolic Diseases, Shanghai Institute of Endocrine and Metabolic Diseases, Ruijin Hospital, Shanghai Jiao Tong University School of Medicine, Shanghai 200025, China; Shanghai National Clinical Research Center for Metabolic Diseases, Key Laboratory for Endocrine and Metabolic Diseases of the National Health Commission of the PR China, Shanghai National Center for Translational Medicine, Shanghai 200025, China; Department of Endocrine and Metabolic Diseases, Shanghai Institute of Endocrine and Metabolic Diseases, Ruijin Hospital, Shanghai Jiao Tong University School of Medicine, Shanghai 200025, China; Shanghai National Clinical Research Center for Metabolic Diseases, Key Laboratory for Endocrine and Metabolic Diseases of the National Health Commission of the PR China, Shanghai National Center for Translational Medicine, Shanghai 200025, China; Department of Endocrine and Metabolic Diseases, Shanghai Institute of Endocrine and Metabolic Diseases, Ruijin Hospital, Shanghai Jiao Tong University School of Medicine, Shanghai 200025, China; Shanghai National Clinical Research Center for Metabolic Diseases, Key Laboratory for Endocrine and Metabolic Diseases of the National Health Commission of the PR China, Shanghai National Center for Translational Medicine, Shanghai 200025, China; Shanghai Institute of Nutrition and Health, University of Chinese Academy of Sciences, Chinese Academy of Sciences, Shanghai 200031, China; Department of Endocrine and Metabolic Diseases, Shanghai Institute of Endocrine and Metabolic Diseases, Ruijin Hospital, Shanghai Jiao Tong University School of Medicine, Shanghai 200025, China; Shanghai National Clinical Research Center for Metabolic Diseases, Key Laboratory for Endocrine and Metabolic Diseases of the National Health Commission of the PR China, Shanghai National Center for Translational Medicine, Shanghai 200025, China; Department of Endocrine and Metabolic Diseases, Shanghai Institute of Endocrine and Metabolic Diseases, Ruijin Hospital, Shanghai Jiao Tong University School of Medicine, Shanghai 200025, China; Shanghai National Clinical Research Center for Metabolic Diseases, Key Laboratory for Endocrine and Metabolic Diseases of the National Health Commission of the PR China, Shanghai National Center for Translational Medicine, Shanghai 200025, China; Department of Endocrine and Metabolic Diseases, Shanghai Institute of Endocrine and Metabolic Diseases, Ruijin Hospital, Shanghai Jiao Tong University School of Medicine, Shanghai 200025, China; Shanghai National Clinical Research Center for Metabolic Diseases, Key Laboratory for Endocrine and Metabolic Diseases of the National Health Commission of the PR China, Shanghai National Center for Translational Medicine, Shanghai 200025, China; Department of Endocrine and Metabolic Diseases, Shanghai Institute of Endocrine and Metabolic Diseases, Ruijin Hospital, Shanghai Jiao Tong University School of Medicine, Shanghai 200025, China; Shanghai National Clinical Research Center for Metabolic Diseases, Key Laboratory for Endocrine and Metabolic Diseases of the National Health Commission of the PR China, Shanghai National Center for Translational Medicine, Shanghai 200025, China; Department of Endocrine and Metabolic Diseases, Shanghai Institute of Endocrine and Metabolic Diseases, Ruijin Hospital, Shanghai Jiao Tong University School of Medicine, Shanghai 200025, China; Shanghai National Clinical Research Center for Metabolic Diseases, Key Laboratory for Endocrine and Metabolic Diseases of the National Health Commission of the PR China, Shanghai National Center for Translational Medicine, Shanghai 200025, China; Department of Endocrine and Metabolic Diseases, Shanghai Institute of Endocrine and Metabolic Diseases, Ruijin Hospital, Shanghai Jiao Tong University School of Medicine, Shanghai 200025, China; Shanghai National Clinical Research Center for Metabolic Diseases, Key Laboratory for Endocrine and Metabolic Diseases of the National Health Commission of the PR China, Shanghai National Center for Translational Medicine, Shanghai 200025, China; Institute of Metabolism and Regenerative Medicine, Shanghai Sixth People’s Hospital Affiliated to Shanghai Jiao Tong University School of Medicine, Shanghai 200235, China; Department of Endocrine and Metabolic Diseases, Shanghai Institute of Endocrine and Metabolic Diseases, Ruijin Hospital, Shanghai Jiao Tong University School of Medicine, Shanghai 200025, China; Shanghai National Clinical Research Center for Metabolic Diseases, Key Laboratory for Endocrine and Metabolic Diseases of the National Health Commission of the PR China, Shanghai National Center for Translational Medicine, Shanghai 200025, China; Department of Endocrine and Metabolic Diseases, Shanghai Institute of Endocrine and Metabolic Diseases, Ruijin Hospital, Shanghai Jiao Tong University School of Medicine, Shanghai 200025, China; Shanghai National Clinical Research Center for Metabolic Diseases, Key Laboratory for Endocrine and Metabolic Diseases of the National Health Commission of the PR China, Shanghai National Center for Translational Medicine, Shanghai 200025, China; Shanghai Institute of Biochemistry and Cell Biology, Center for Excellence in Molecular Cell Science, Chinese Academy of Sciences, Shanghai 200031, China; Department of Endocrine and Metabolic Diseases, Shanghai Institute of Endocrine and Metabolic Diseases, Ruijin Hospital, Shanghai Jiao Tong University School of Medicine, Shanghai 200025, China; Shanghai National Clinical Research Center for Metabolic Diseases, Key Laboratory for Endocrine and Metabolic Diseases of the National Health Commission of the PR China, Shanghai National Center for Translational Medicine, Shanghai 200025, China; Shanghai Institute of Nutrition and Health, University of Chinese Academy of Sciences, Chinese Academy of Sciences, Shanghai 200031, China; Department of Endocrine and Metabolic Diseases, Shanghai Institute of Endocrine and Metabolic Diseases, Ruijin Hospital, Shanghai Jiao Tong University School of Medicine, Shanghai 200025, China; Shanghai National Clinical Research Center for Metabolic Diseases, Key Laboratory for Endocrine and Metabolic Diseases of the National Health Commission of the PR China, Shanghai National Center for Translational Medicine, Shanghai 200025, China; Department of Endocrine and Metabolic Diseases, Shanghai Institute of Endocrine and Metabolic Diseases, Ruijin Hospital, Shanghai Jiao Tong University School of Medicine, Shanghai 200025, China; Shanghai National Clinical Research Center for Metabolic Diseases, Key Laboratory for Endocrine and Metabolic Diseases of the National Health Commission of the PR China, Shanghai National Center for Translational Medicine, Shanghai 200025, China; Department of Endocrine and Metabolic Diseases, Shanghai Institute of Endocrine and Metabolic Diseases, Ruijin Hospital, Shanghai Jiao Tong University School of Medicine, Shanghai 200025, China; Shanghai National Clinical Research Center for Metabolic Diseases, Key Laboratory for Endocrine and Metabolic Diseases of the National Health Commission of the PR China, Shanghai National Center for Translational Medicine, Shanghai 200025, China; Department of Endocrine and Metabolic Diseases, Shanghai Institute of Endocrine and Metabolic Diseases, Ruijin Hospital, Shanghai Jiao Tong University School of Medicine, Shanghai 200025, China; Shanghai National Clinical Research Center for Metabolic Diseases, Key Laboratory for Endocrine and Metabolic Diseases of the National Health Commission of the PR China, Shanghai National Center for Translational Medicine, Shanghai 200025, China

**Keywords:** SPARC, insulin sensitivity, the Mediterranean diet, caloric restriction, lipidomics, precision nutrition

## Abstract

Precision nutrition is pivotal to preventing cardiometabolic diseases. However, almost no single blood biomarker capable of predicting the metabolic benefits of specific dietary patterns has yet been identified. Here, we revealed the associations of plasma levels of the secreted protein acidic and rich in cysteine (SPARC), an inflammatory factor highly expressed in fat tissues, and insulin sensitivity improvement in a 6-month randomized controlled, calorie-restricted feeding trial recruiting 235 Chinese adults with overweight/obesity and prediabetes: the Mediterranean diet (MD) group (*n *= 81), the traditional Jiangnan diet (TJD) group (*n *= 81), and the control diet (CD) group (*n *= 73). The 1-h post-glucose loading plasma SPARC levels (SPARC-1H) decreased significantly from baseline to 3 months and 6 months in the MD group, whereas no significant changes were observed in the TJD or CD groups. Further analyses revealed that the individuals with higher baseline SPARC-1H levels were associated with fewer improvements in fasting insulin (β ± SE: 1.54 ± 0.43; *P *= 0.002), fasting glucose (0.10 ± 0.04; *P *= 0.049), the homeostasis model assessment of insulin resistance (HOMA-IR, 0.47 ± 0.12; *P *= 0.0005), and the homeostasis model assessment of β-cell function (HOMA-β, 7.63 ± 3.27; *P *= 0.047) after 6 months in the MD group. Moreover, baseline SPARC-1H levels were positively correlated with changes in lipidomic profiles, including three alkenylphosphatidylethanolamines, which potentially mediate the cardiometabolic benefits of MD. No significant associations were observed in the other two diet groups. Our findings suggest postprandial SPARC as a predictor for the metabolic benefits of MD, offering a potential biomarker for individualized nutrition intervention against cardiometabolic diseases.

## Introduction

Unhealthy diet is a prominent contributor to the epidemic of chronic diseases including obesity, type 2 diabetes, and cardiovascular disease (CVD) worldwide [1–3]. Several healthy dietary patterns have been recommended for the prevention and management of cardiometabolic diseases, however, a one-size-fits-all dietary prescription has become obsolete, as the responses to diets vary among individuals [4]. Consequently, the concept of precision nutrition has gained momentum, aiming to provide tailored nutrition recommendations based on their genetics, microbiome, socio­economic, and environmental factors, along with other unique characteristics [5].

Mediterranean diet (MD), which is characterized by abundant vegetables and fruits, whole grains, olive oil, marine fish, low-to-­moderate intake of wine, milk and dairy products, and little red meat [6], has been demonstrated to reduce the risk of cardiometabolic diseases [7–10]. Both the American Heart Association/American College of Cardiology and the American Diabetes Association have recommended the dietary formula as a vital lifestyle approach in the prevention and management of CVD and diabetes [11–13]. Several studies demonstrate that the MD yields benefit effects on metabolites [14], circulating factors [15], inflammatory biomarkers [16], and gut microbiome [17, 18]. Furthermore, it has been shown that genetic factor, lifestyles, and the gut microbiome could modulate the impact of MD on cardiometabolic disease development [19–21]; as a result, the responses of MD vary among different individuals. However, the factors that predict the metabolic response to an MD are typically reflected in omics-level features (such as polygenic risk score or gut flora mixture) [22], and no single indicator has yet been identified to date, which limits the clinical application of the precision nutrition. In addition, although quite a few biomarkers change during the MD intervention, little is known whether and how biomarkers are involved in the association between the MD and cardiometabolic risk reduction.

The secreted protein acidic and rich in cysteine (SPARC), also known as osteonectin or BM-40 [23], is a secreted protein in the extracellular matrix, playing pivotal role in angiogenesis, collagen processing, tumorigenesis, and inflammation [24–26]. Circulating SPARC is reported elevated in patients with obesity, diabetes, and CVD, and correlates with insulin resistance and inflammatory factors [27–29]. Recently, the expression of SPARC, as an important and novel adipokine, was reported to be suppressed in adipose tissue by caloric restriction (CR) in a calorie-restricted clinical trial (CALERIE-II) [30]. Ablation of *SPARC* gene expression specifically in adipose tissue could enhance the glucose metabolism through the amelioration of inflammation [31, 32]. However, the local roles of SPARC in adipose tissue may differ from its global influences in circulation. Few clinical studies have evaluated the circulating SPARC in a calorie-restricted diet intervention.

In our previous clinical trial, we assessed the effects of three diet patterns, including the MD, on body weight loss and glucose improvement [33]. In the present study, we systematically measured the plasma SPARC levels at three time points (0 h, 1 h, and 2 h) during the 75-g oral glucose tolerance tests (OGTT) at baseline, 3-month, and 6-month intervention, respectively. We investigated the changes of the circulating SPARC during OGTT in CR and ­examined the association between circulating SPARC and the effects of the MD on insulin sensitivity. Particularly, we explored the possible mechanisms of lipidomics underlying the distinct response to the MD among individuals.

## Results

### Baseline characteristics

**Figure 1:**
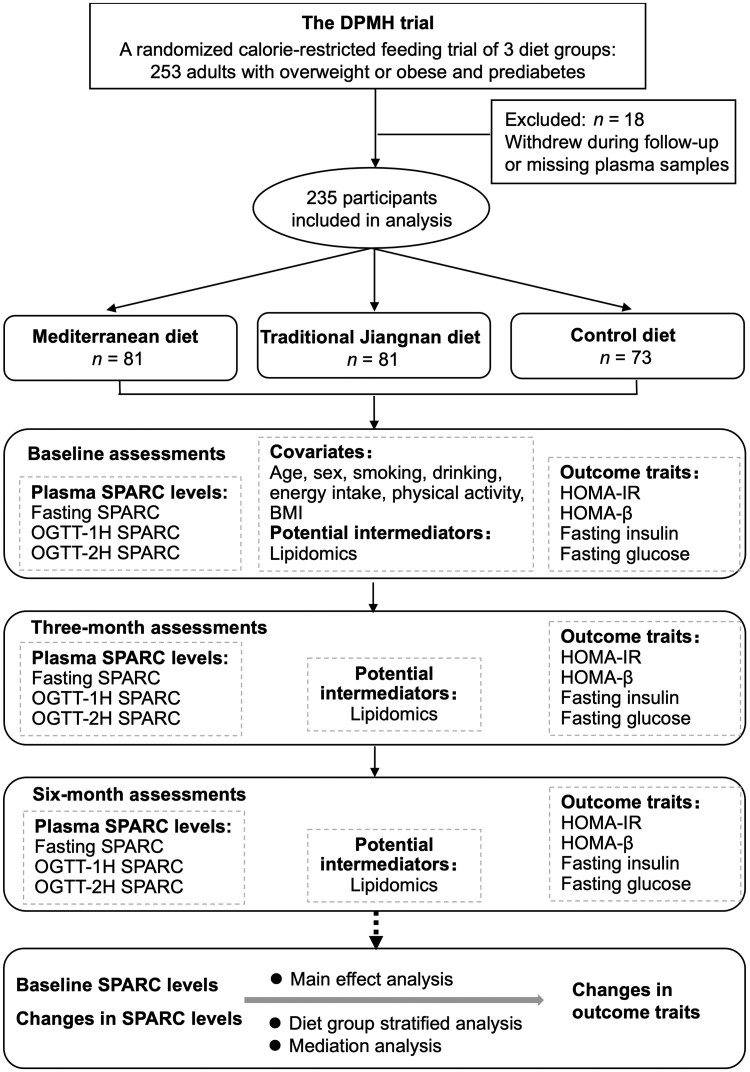
Flow diagram demonstrating the study design.

The study flow for the *post hoc* analysis of the Dietary Pattern and Metabolic Health (DPMH) trial is shown in Fig. 1. A total of 235 participants underwent a 25% calorie-restricted feeding dietary intervention for 6 months, including 81 in the MD group, 81 in the traditional Jiangnan diet (TJD) group, and 73 in the low-plant-content CD group. We carried out OGTT after overnight fasting at baseline, 3 months, and 6 months across the study. The levels of fasting plasma SPARC, SPARC at OGTT-1H (SPARC-1H), and SPARC at OGTT-2H (SPARC-2H) were measured.

Clinical and biochemical characteristics according to the three diet groups are shown in Table 1. We found no significant differences in age, sex, lifestyle, body mass index (BMI), fasting glucose, fasting insulin, the homeostasis model assessment of insulin resistance (HOMA-IR), and the homeostasis model assessment of β-cell function (HOMA-β) (all *P *> 0.1) among the MD, TJD, and CD groups.

**Table 1:** Baseline clinical characteristics in the three diet groups.

Characteristics	**MD ** **(*n *= 81)**	**TJD ** **(*n *= 81)**	**CD ** **(*n *= 73)**	*P* value
**Male, *n* (%)**	68 (84.0)	69 (85.2)	65 (89.0)	0.64^*^
**Age, years**	38.0 ± 8.6	37.3 ± 8.7	38.4 ± 9.0	0.71
**Current smoker, *n* (%)**	16 (19.8)	11 (13.6)	20 (27.4)	0.10^*^
**Drinking, days/week**	4.2 ± 0.5	4.3 ± 0.5	4.2 ± 0.6	0.35
**Total energy intake**				
** Male, kcal/day**	1961 ± 414	2015 ± 516	1966 ± 472	0.76
** Female, kcal/day**	1893 ± 412	1738 ± 226	1701 ± 237	0.33
**Physical activity, min/week**	1607 ± 1595	1769 ± 2000	1778 ± 1481	0.78
**BMI, kg/m^2^**	26.6 ± 3.2	26.8 ± 3.0	26.8 ± 3.1	0.89
**HOMA-IR**	4.13 ± 2.80	4.63 ± 3.47	4.40 ± 2.37	0.56
**HOMA-β**	109.5 ± 66.5	130.4 ± 79.0	118.0 ± 58.4	0.16
**Fasting insulin, µIU/mL**	14.72 ± 9.60	16.84 ± 11.67	15.69 ± 7.96	0.40
**Fasting glucose, mmol/L**	6.25 ± 0.73	6.10 ± 0.52	6.24 ± 0.80	0.30
**hs-CRP, mg/L**	1.50 ± 1.61	2.15 ± 4.99	1.68 ± 2.38	0.45
**Fasting SPARC, ng/mL**	44.17 ± 24.05	42.86 ± 22.46	40.31 ± 24.79	0.60
**SPARC-1H, ng/mL**	63.52 ± 49.90	58.77 ± 37.65	52.03 ± 28.31	0.21
**SPARC-2H, ng/mL**	64.81 ± 48.55	64.49 ± 49.04	54.44 ± 39.19	0.30

Data are presented as mean ± SD or *n* (%). ^*^P value was calculated using one-way ANOVA or χ^2^ tests.

Abbreviations: BMI, body mass index; CD, control diet; hs-CRP, hypersensitive C-reactive protein; MD, Mediterranean diet; SD, standard deviation; TJD, traditional Jiangnan diet.

### SPARC changes during OGTT

**Figure 2:**
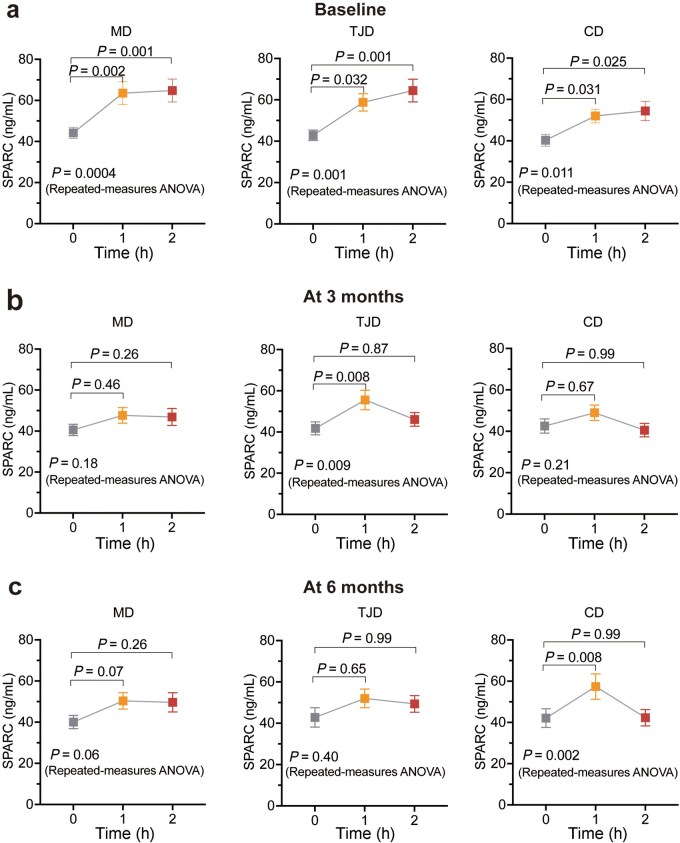
Change patterns of SPARC levels during OGTT at baseline, 3 months, and 6 months. (a–c) Plasma SPARC levels are increased after 1-h and 2-h stimulation of oral glucose intake in the three groups at baseline (a), while showing increasing trends at 3 months (b) and 6 months (c). Comparations were calculated by repeated-measures ANOVA, adjusting for sex, baseline age, baseline BMI, total energy intake, physical activity, smoking, and drinking. *Post hoc* pairwise comparisons between 0 h and 1 or 2 h were conducted with Bonferroni adjustment. For SPARC at baseline, *n *= 81 for the MD group, *n *= 81 for the TJD group, and *n *= 73 for the CD group. For SPARC at 3 months, *n *= 72 for the MD group, *n* = 73 for the TJD group, and *n *= 65 for the CD group. For SPARC at 6 months, *n *= 66 for the MD group, *n *= 61 for the TJD group, and *n *= 57 for the CD group.

We firstly analyzed SPARC levels during OGTT. Intriguingly, plasma SPARC levels increased 15.78 ng/mL (95% confidence interval [CI]: 10.19−21.38) at OGTT-1H and 18.95 ng/mL (95% CI: 12.21–25.69) at OGTT-2H, respectively, after glucose intake in all participants at baseline (Supplementary Fig. S1a). At 3 months and 6 months, plasma SPARC levels showed significant increase at OGTT-1H and then declined to the baseline levels at OGTT-2H in all participants (Supplementary Fig. S1b and c). At baseline, similar changes of plasma SPARC levels during OGTT were observed across the three diet groups (Fig. 2a). SPARC-1H levels were significantly higher than fasting levels in all groups (19.36 ng/mL [95% CI: 8.14–30.57] in the MD group; 15.90 ng/mL [95% CI: 6.69–25.12] in the TJD group; 11.71 ng/mL [95% CI: 3.00–20.42] in the CD group, respectively). The SPARC-2H levels showed a slight increase compared with that of SPARC-1H and a more marked elevation than fasting SPARC (20.64 ng/mL [95% CI: 8.89–32.39] in the MD group; 21.63 ng/mL [95% CI: 9.55–33.71] in the TJD group; 14.13 ng/mL [95% CI: 2.45–25.80] in the CD group, respectively). Of note, there were no differences in fasting SPARC, SPARC-1H, and SPARC-2H levels among the three groups at baseline (*P * = 0.60, 0.21, 0.30, respectively; Table 1). At 3 months and 6 months, plasma SPARC levels showed an increasing trend during OGTT (Fig. 2b and c).

### SPARC-1H levels during the intervention differ across the diet groups

**Figure 3:**
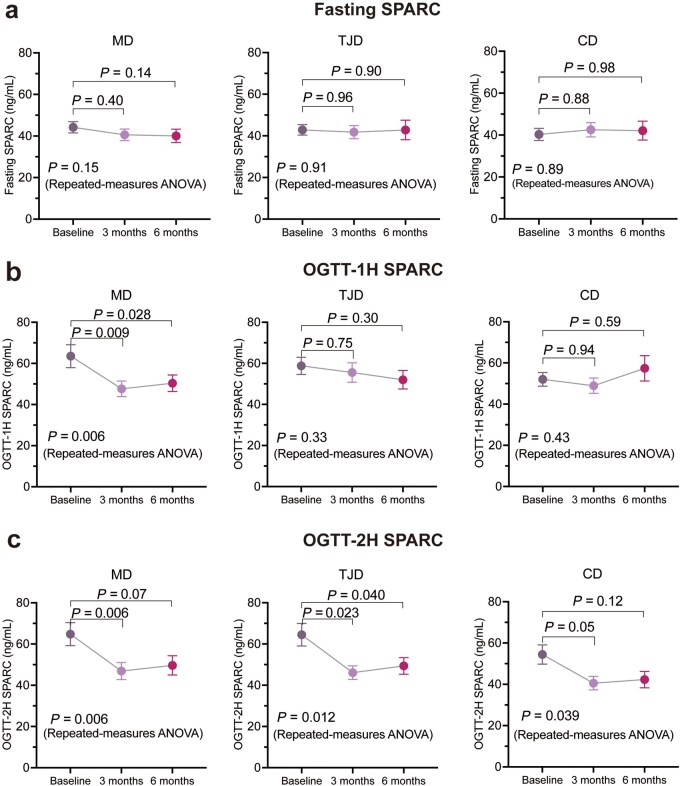
Changes of SPARC during dietary intervention across groups. (a−c) Fasting plasma SPARC levels (a), OGTT-1H plasma SPARC (SPARC-1H) levels (b), and OGTT-2H plasma SPARC (SPARC-2H) levels (c) among baseline, 3 months, and 6 months in all three groups. *P* values among the three stages of the study were calculated by repeated-measures ANOVA, adjusting for sex, baseline age, baseline BMI, total energy intake, physical activity, smoking, and drinking. *P* values between baseline and 3 months or 6 months were calculated by *post hoc* pairwise comparisons with Bonferroni adjustment. For fasting SPARC, *n *= 81 for the MD group, *n *= 81 for the TJD group, and *n *= 73 for the CD group. For SPARC-1H, *n *= 81 for the MD group, *n *= 81 for the TJD group, and *n *= 73 for the CD group. For SPARC-2H, *n *= 79 for the MD group, *n *= 81 for the TJD group, and *n *= 72 for the CD group.

Fasting plasma SPARC concentration did not change obviously at 3-month or 6-month follow-ups in all three diet groups (Fig. 3a). Intriguingly, only in the MD group, plasma SPARC-1H levels decreased significantly after 3-month (*P *= 0.009) and 6-month (*P *= 0.028) CR (Fig. 3b), while no significant changes of SPARC-1H levels were found in the TJD or CD group (Fig. 3b). In addition, we observed that plasma SPARC-2H levels declined during 6-month intervention in all three diet groups (*P *= 0.006 for the MD group; *P *= 0.012 for the TJD group; *P *= 0.039 for the CD group) (Fig. 3c).

### Baseline SPARC-1H levels predict cardiometabolic changes

Participants lost 5.6 kg body weight on average and improved insulin resistance after 6-month dietary intervention (Supplementary Table S1), as we previously reported [33]. Next, we tested whether baseline SPARC levels were associated with changes in cardiome­tabolic characteristics. Among all participants, lower baseline SPARC-1H levels were associated with a greater reduction in HOMA-IR (*P *= 0.027) and fasting insulin (*P *= 0.043) from baseline to 6 months, after adjusting for sex, baseline age, BMI, smoking and alcohol drinking, physical activity, total energy intake, baseline values for each respective outcome, and diet groups (­Supplementary Table S2). There was an interaction between baseline SPARC-1H levels and diet groups (Supplementary Table S2). We did not observe any significant association between baseline SPARC-1H levels and the changes in HOMA-IR, HOMA-β, fasting insulin, or fasting glucose from baseline to 3 months. In addition, there were no significant differences between fasting SPARC or SPARC-2H levels and the changes in HOMA-IR, HOMA-β, fasting insulin, or fasting ­glucose (Supplementary Tables S3 and S4).

We then analyzed the above mentioned associations stratified by the three diet groups (Table 2). No significant differences were observed during the 3-month follow-up in all three groups. Notably, baseline SPARC-1H levels were positively associated with the changes in HOMA-IR (*P *= 0.0005), HOMA-β (*P *= 0.047), fasting insulin (*P *= 0.002), and fasting glucose (*P *= 0.049) from baseline to 6 months, specifically in the MD group, after adjustment for sex, baseline age, BMI, smoking and alcohol drinking, physical activity, total energy intake, and baseline values for each respective outcome. There were no significant differences between SPARC-1H levels and HOMA-IR, HOMA-β, fasting insulin, and fasting glucose in the TJD or CD group.

**Table 2:** The association between the plasma SPARC-1H levels at baseline and the metabolic changes from baseline to 3 and 6 months in the three groups.

		Baseline SPARC-1H					
		MD		TJD		CD	
		**β** ± **SE**	*P* value	**β** ± **SE**	*P* value	**β** ± **SE**	*P* value
**Metabolic changes from baseline to 3 months**							
**Model 1**	HOMA-IR	0.25 ± 0.23	0.29	−0.22 ± 0.54	0.69	−0.10 ± 0.15	0.49
	HOMA-β	2.52 ± 4.98	0.61	−2.22 ± 8.46	0.79	−2.09 ± 3.05	0.50
	Fasting insulin, µIU/mL	0.78 ± 0.69	0.26	−0.58 ± 1.63	0.72	−0.31 ± 0.46	0.51
	Fasting glucose, mmol/L	0.09 ± 0.06	0.17	−0.03 ± 0.06	0.64	−0.05 ± 0.06	0.35
**Model 2**	HOMA-IR	0.20 ± 0.25	0.43	−0.33 ± 0.61	0.59	0.12 ± 0.15	0.43
	HOMA-β	2.68 ± 5.21	0.61	0.17 ± 9.31	0.99	0.33 ± 3.32	0.92
	Fasting insulin, µIU/mL	0.68 ± 0.73	0.36	−0.75 ± 1.84	0.68	0.30 ± 0.46	0.52
	Fasting glucose, mmol/L	0.06 ± 0.07	0.40	−0.05 ± 0.06	0.39	0.03 ± 0.06	0.68
**Metabolic changes from baseline to 6 months**							
**Model 1**	HOMA-IR	0.47 ± 0.12	**0.0002**	0.46 ± 0.28	0.10	−0.01 ± 0.19	0.94
	HOMA-β	7.63 ± 3.27	**0.023**	6.20 ± 6.78	0.37	−0.78 ± 5.40	0.89
	Fasting insulin, µIU/mL	1.54 ± 0.43	**0.0007**	1.38 ± 0.94	0.15	−0.04 ± 0.67	0.95
	Fasting glucose, mmol/L	0.10 ± 0.04	**0.013**	0.02 ± 0.05	0.69	−0.02 ± 0.05	0.73
**Model 2**	HOMA-IR	0.45 ± 0.12	**0.0005**	0.53 ± 0.30	0.08	0.03 ± 0.21	0.88
	HOMA-β	6.91 ± 3.40	**0.047**	7.65 ± 7.38	0.31	−0.94 ± 6.03	0.88
	Fasting insulin, µIU/mL	1.46 ± 0.44	**0.002**	1.56 ± 1.01	0.13	0.06 ± 0.74	0.93
	Fasting glucose, mmol/L	0.09 ± 0.04	**0.049**	0.02 ± 0.06	0.69	0.04 ± 0.05	0.47

Data are presented as β ± SE. Statistically significant (*P* values < 0.05) values were highlighted in bold. The associations between the plasma SPARC-1H levels at baseline and the changes of HOMA-IR, HOMA-β, fasting insulin, and fasting glucose (the levels of clinical characteristics at 3 or 6 months minus those at baseline) were assessed by using GLM, after z-score normalization for baseline plasma SPARC-1H levels.

Model 1: adjusted for sex, and baseline age, BMI, and respective values for each outcome.

Model 2: based on Model 1 and further adjusted for total energy intake, physical activity, smoking, and drinking.

We further estimated the associations between baseline fasting SPARC or SPARC-2H levels and metabolic changes. Fasting SPARC levels showed a positive correlation with the changes of HOMA-IR and fasting insulin from baseline to 6 months in the TJD group (Supplementary Table S5), while no additional significance was found between fasting SPARC or SPARC-2H levels and the changes in HOMA-IR, HOMA-β, fasting insulin, or fasting glucose in three groups (Supplementary Tables S5 and S6).

### Associations between baseline SPARC-1H levels and the lipidomic changes during the intervention

**Figure 4:**
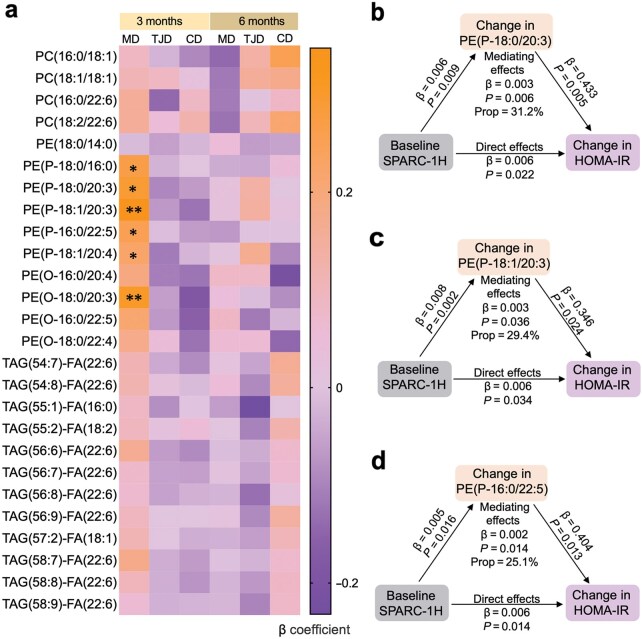
The association between the plasma SPARC-1H levels at baseline and the changes of lipids from baseline to 3 and 6 months. (a) Heatmap showing that plasma SPARC-1H levels at baseline are positively correlated with the changes of five PE(P)s and one PE(O) from baseline to 3-month in the MD group using GLM method. SPARC-1H levels were normalized by z-score method. (b–d) Mediation analysis showing the mediating effects of PE(P)s between the associations of the baseline SPARC-1H levels and the metabolic changes. All analyses were adjusted for age, sex, baseline BMI, respective baseline values of lipids, total energy intake, physical activity, smoking and drinking, education, and waist. ^*^P < 0.05; ^**^P < 0.01

In our recent study, we evaluated blood lipidomic signatures in the three diet groups and found that 26 out of 364 lipidomic species were altered during the intervention. The MD group showed a distinct lipid profile when compared with the other two groups, and alkenylphosphatidylethanolamines (PE(P)s) and alkylphosphatidylethanolamine (PE(O)s), which were possibly related to the red meat intake, decreased significantly in the MD intervention [34]. We then analyzed the relationship between baseline SPARC-1H levels and the changes of the 26 lipids. There was a positive association between baseline SPARC-1H levels and the changes in five PE(P)s and one PE(O) from baseline to 3 months in the MD group (Fig. 4a; Table 3). In the MD group, the baseline to 3-month changes of PE(P-18:0/20:3), PE(P-18:1/20:3), and PE(P-16:0/22:5) showed mediating effects between the baseline SPARC-1H levels and the changes in HOMA-IR and fasting insulin from baseline to 6 months (Fig. 4b−d; Supplementary Table S7).

**Table 3:** The association between the plasma SPARC-1H levels at baseline and the changes in lipids from baseline to 3 and 6 months in the three groups.

	Baseline SPARC-1H								
	MD			TJD			CD		
	**β** ± **SE**	*P* value	*P*_FDR_	**β** ± **SE**	*P* value	*P*_FDR_	**β** ± **SE**	*P* value	*P*_FDR_
**Changes in lipids from baseline to 3 months**									
**PE(P-18:0/16:0)**	0.27 ± 0.11	**0.017**	0.04	−0.03 ± 0.11	0.80	0.90	0.001 ± 0.10	0.996	0.996
**PE(P-18:0/20:3)**	0.29 ± 0.11	**0.011**	0.03	−0.09 ± 0.13	0.45	0.82	−0.07 ± 0.11	0.56	0.72
**PE(P-18:1/20:3)**	0.35 ± 0.11	**0.003**	0.02	−0.07 ± 0.12	0.55	0.82	−0.13 ± 0.11	0.26	0.53
**PE(P-16:0/22:5)**	0.25 ± 0.11	**0.022**	0.04	−0.01 ± 0.12	0.94	0.94	−0.06 ± 0.11	0.55	0.72
**PE(P-18:1/20:4)**	0.22 ± 0.11	**0.043**	0.06	−0.11 ± 0.11	0.30	0.82	−0.02 ± 0.11	0.83	0.94
**PE(O-16:0/20:4)**	0.19 ± 0.11	0.10	0.10	−0.11 ± 0.08	0.20	0.82	−0.12 ± 0.11	0.28	0.53
**PE(O-18:0/20:3)**	0.31 ± 0.11	**0.005**	0.02	−0.06 ± 0.09	0.52	0.82	−0.19 ± 0.11	0.10	0.53
**PE(O-16:0/22:5)**	0.21 ± 0.11	0.06	0.07	−0.07 ± 0.11	0.54	0.82	−0.16 ± 0.12	0.17	0.53
**PE(O-18:0/22:4)**	0.17 ± 0.10	0.08	0.09	0.05 ± 0.13	0.72	0.90	−0.13 ± 0.12	0.29	0.53
**Changes in lipids from baseline to 6 months**									
**PE(P-18:0/16:0)**	−0.04 ± 0.10	0.71	0.95	−0.04 ± 0.14	0.74	0.96	0.04 ± 0.11	0.70	0.99
**PE(P-18:0/20:3)**	0.02 ± 0.10	0.85	0.95	0.14 ± 0.13	0.29	0.76	0.001 ± 0.12	0.99	0.99
**PE(P-18:1/20:3)**	0.02 ± 0.10	0.80	0.95	0.14 ± 0.13	0.26	0.76	0.02 ± 0.12	0.88	0.99
**PE(P-16:0/22:5)**	−0.07 ± 0.10	0.48	0.95	−0.003 ± 0.12	0.98	0.98	−0.002 ± 0.12	0.98	0.99
**PE(P-18:1/20:4)**	0.01 ± 0.09	0.95	0.95	0.17 ± 0.14	0.25	0.76	−0.09 ± 0.13	0.50	0.99
**PE(O-16:0/20:4)**	0.08 ± 0.10	0.43	0.95	0.08 ± 0.13	0.55	0.88	−0.21 ± 0.13	0.11	0.99
**PE(O-18:0/20:3)**	0.04 ± 0.10	0.72	0.95	−0.01 ± 0.14	0.93	0.98	−0.09 ± 0.11	0.44	0.99
**PE(O-16:0/22:5)**	0.08 ± 0.10	0.41	0.95	−0.12 ± 0.12	0.34	0.76	−0.02 ± 0.11	0.85	0.99
**PE(O-18:0/22:4)**	0.06 ± 0.08	0.49	0.95	0.06 ± 0.11	0.59	0.88	−0.14 ± 0.14	0.31	0.99

Data are presented as β ± SE. Statistically significant (*P* values < 0.05) values were highlighted in bold. The associations between the baseline plasma SPARC-1H levels (z-score normalization) and the changes of lipids from baseline to 3 or 6 months were assessed by using GLM, adjusting for sex, baseline age, baseline BMI, respective baseline values of lipids, total energy intake, physical activity, smoking and drinking, education, and waist. *P*_FDR_ was calculated by FDR correction using the Benjamini−Hochberg method.

## Discussion

To the best of our knowledge, it is the first study that we described a change pattern of circulating SPARC levels during calorie-restricted feeding intervention, and in further, extensively assessed the association between the circulating SPARC levels and the cardiometabolic outcome changes under different dietary patterns in overweight/obese adults with prediabetes. Here, we identified that in the MD group but not in the TJD or CD group, baseline SPARC-1H levels were positively associated with the changes in HOMA-IR, HOMA-β, fasting insulin, and fasting glucose over the 6-month trial period. In addition, we linked SPARC-1H levels to the changes of lipidomics after adherence to MD.

In this study, we provided a systematic description of the pattern of SPARC levels during OGTT and its change trajectory during CR intervention for the first time. We discovered that SPARC levels increased rapidly after oral glucose intake and SPARC-1H levels declined significantly from baseline to 3 months and 6 months only in the MD group, which has not been previously reported. A recent study by Ryu *et al.* showed that 14% sustained CR (CALERIE-II clinical trial) reduced the expression of SPARC in adipose tissue according to the RNA sequencing analyses, and similar results were observed in mice [30, 31]. Another study observed a significant reduction of fasting serum SPARC levels after metabolic surgery in obese patients [35]. However, no studies have explored the changes of postprandial SPARC. We did not observe appreciable changes of fasting SPARC in our study. Intriguingly, plasma SPARC-1H levels showed a significant decrease from baseline to 3 months and 6 months in the MD group, while no such changes were observed in the TJD or CD group. Our finding suggests that fasting SPARC and SPARC-1H may have different biological functions and/or clinical implications.

In the current study, we showed that SPARC-1H predicts improvements in glucose homeostasis in the MD intervention under a context of 25% CR, which is not observed in the TJD or CD group. It has been well known that CR alone exerts a favorable influence on improving insulin sensitivity, which aligns with our recent findings [33, 36, 37]. A meta-analysis of 50 studies and 534,906 individuals revealed that adherence to the MD plays a protective role in metabolic syndrome as well as its components such as glucose and HOMA-IR [7]. In further, adipose-derived and circulating SPARC exhibit a positive association with HOMA-IR in humans [38, 39]. In mouse models, adipocyte-specific *Sparc* gene knockout improves glucose tolerance and insulin sensitivity [31]. Mechanistically, downregulation of SPARC can alleviate inflammation in adipose tissue via the nucleotide-binding domain, leucine-rich-repeat containing family, pyrin domain-containing 3 (NLRP3) inflammasome and the c-Jun N-terminal kinase (JNK)/p38 mitogen-activated protein kinase (MAPK) signaling pathway, and thus can protect against diet-induced obesity and improve metabolic health [31]. Nonetheless, little is known about the linkage of blood SPARC and systemic insulin resistance in the MD. Unanticipatedly, our findings demonstrated that participants with lower baseline SPARC-1H levels may experience more improvement in insulin sensitivity and less risk of diabetes mellitus. We reported for the first time that a single blood biomarker, SPARC-1H, modulates the protective association between the MD and glucose homeostasis.

Postprandial SPARC, rather than fasting SPARC, predicts improvements in glucose homeostasis during diet intervention. We speculated about some possible mechanisms. Circulating SPARC is predominantly secreted by adipose tissue [40]. Mature adipocytes, particularly a subtype of mature adipocytes with large size marked by perilipin-1 (PLIN1) and PLIN4, are major sources of SPARC secretion [38, 41]. These adipocytes play critical roles in maintaining glucose and lipid metabolic homeostasis and in the development of insulin resistance. Following glucose stimulation or food intake, adipokines (such as leptin) and insulin are markedly secreted. Both *in vivo* and *in vitro* studies have demonstrated that leptin and insulin can promote SPARC secretion from adipocytes in a dose-dependent manner [38, 42]. These findings suggest that SPARC may function as a novel adipokine that responds to nutrient stimuli and participates in the postprandial regulation of glucose and lipid metabolism. In this context, like basal and glucose-responsive secretion of insulin, fasting SPARC may represent the basal secretion capacity of adipocytes, in contrast, SPARC-1H reflects the responsive capacity under external nutrition stress and consequently shows unique roles in disease prediction. Notably, during the 6-month MD intervention, fasting SPARC did not exhibit sign­ificant changes, whereas SPARC-1H decreased significantly, highlighting that SPARC-1H is a more sensitive indicator of dietary intervention response. Thus, SPARC-1H acts as a more sensitive biomarker than fasting SPARC in predicting the metabolic improvements of dietary intervention.

We provide the evidence that SPARC serves as a blood predictor of CR-related metabolic benefits specifically in the MD but not in the TJD or CD. We suggest that there are several possibilities contributing to this dietary pattern specificity. First, the MD is characterized by olive oil, nuts, marine fish, and low red meat, which make its lipidomic signatures distinct from other dietary patterns [14, 34]. Red meat contains a certain amount of arachidonic acid (20:4n-6) and plasmalogens, including PE(P)s and PE(O)s [43]. Our recent analysis of plasma lipidomics revealed that there is a significant reduction in PE(P)s and PE(O)s, possibly due to low red meat intake, in the MD group but not in the CD group [34]. C20:3-containing PE(P)s are associated with elevated fasting glucose and deteriorated insulin sensitivity [34]. Of note, C20:3- and C20:4-containing plasmalogens can exacerbate oxidative stress and inflammation which are closely related to the development of cardiovascular and metabolic diseases [44, 45]. Plasmalogens are particularly rich in long-chain saturated fatty acids, which have been shown to activate the GPR120 signaling pathway in adipocytes, leading to the secretion of inflammatory cytokines such as interleukin-6 (IL-6) [46]. Given that SPARC is also an adipocyte-derived adipokine involved in the regulation of adipose tissue inflammation [31], it is conceivable that plasmalogens may modulate SPARC secretion through lipid-sensing pathways that converge on inflammatory or metabolic signaling. On the other hand, SPARC may exert upstream effects on lipid metabolism. A plausible mechanistic hypothesis is that elevated baseline SPARC-1H levels enhance mitochondrial remodeling and oxidative stress responses [47], thereby facilitating adaptive remodeling of plasmalogens under the MD intervention. It should also be noted that 364 lipid species were detected in this study, but a good number of lipids remain undetectable such as phenolic compounds related to olive oil. As a result, there are possibilities that other lipids, in addition to plasmalogens, also mediate the effects of SPARC.

Second, there probably are unique ways of ameliorating inflammation by MD. MD is cooked by extra-virgin olive oil which is not used in TJD or CD. Olive oil, possessing monounsaturated fatty acids and phenolic compounds such as hydroxytyrosol and oleocanthal, has anti-inflammatory and antioxidant activities [48, 49]. As mentioned, plasmalogens, related to red meat which are low in MD, are associated with inflammatory reactions [45]. A cross-sectional study of gestational diabetes mellitus demonstrated that higher concentrations of circulating SPARC are ­associated with hypersensitive C-reactive protein (hs-CRP), an inflammatory factor [50]. Moreover, SPARC facilitates cardiac inflammation by increasing proinflammatory macrophage M1 infiltration in mice [32], and in consistence, mice with adipose tissue-specific deficiency of *Sparc* obtain CR-mimic metabolic and anti-inflammatory benefits [31]. Therefore, it is plausible to suppose that inflammation relief plays a central role in the prediction of cardiometabolic changes in the MD by baseline plasma SPARC-1H.

Third, the gut microbiome after the MD intervention may be different from that of TJD and CD. A recent study showed that the protective effects of the MD against CVD risk, including triglyceride, hs-CRP, and hemoglobin A1c (HbA1c), vary with different microbial compositions [19]. For instance, a reduction in the abundance of *Prevotella copri* enhances the protective association between the MD and the CVD risk. The abundance of microbiome enriched by the MD exhibits a negative correlation with inflammatory markers, including IL-17 and hs-CRP [17], while little is known about the association between SPARC and microbiome, which requires to be further investigated.

It is worth noting that in addition to the roles of SPARC-1H in the MD group, we also found that baseline fasting SPARC levels were positively associated with the changes in HOMA-IR and fasting insulin during the TJD intervention. The divergent roles of SPARC observed across dietary interventions may be attributable to differences in nutrient composition. The TJD diet—characterized by 7% higher carbohydrate and 7% lower fat with fewer anti-inflammatory lipid components compared to the MD—likely induces stronger postprandial insulin excursions and more direct adipose tissue related metabolic response [33]. The predictive effect of fasting SPARC on glycemic improvement may operate through adiposity related pathways, such as adipose tissue remodeling and insulin signaling modulation. These findings indicate that SPARC acts as a context-dependent predictor, whose functional relevance varies with the dietary environment. Because dietary patterns differ markedly in macronutrient composition and bioactive components, they may exert distinct interaction with SPARC, underscoring the importance of considering dietary context when interpreting the predictive or mechanistic roles of SPARC in metabolic regulation.

In conclusion, postprandial plasma SPARC (but not fasting SPARC) effectively and specifically predicts the cardiometabolic benefits of the MD intervention. Individuals with cardiometabolic risks but showing lower SPARC-1H levels achieve greater improvements of insulin sensitivity when adherent to the MD, possibly through interactions with unique lipids.

### Limitations of the study

The strengths of our study include the comprehensive measurements of SPARC levels during OGTT across a 6-month CR dietary intervention using the enzyme-linked immunosorbent assay (ELISA) method. Notably, we conducted an evaluation of the associations of SPARC levels with cardiometabolic outcomes and lipidomic components. Nevertheless, several limitations should be acknowledged in this study. First, the participants included in our study were prediabetic; it remains uncertain whether our findings apply to patients with diabetes, which is required to be verified in future trial studies. Second, the underlying mechanism of SPARC in predicting the MD benefits requires further investigation. Here, we only analyzed the associations of SPARC levels with lipids, while more inflammatory factors and gut microbiome remain to be explored. Last, although circulating SPARC mainly comes from adipose tissues, other tissues such as the muscles, blood vessels, and gut also secrete SPARC. Up to now, it remains unclear about the exact source(s) of SPARC after acute glucose stimulation.

## Materials and methods

### Study design and participants

The DPMH trial was a parallel-arm randomized controlled feeding trial that assessed the effects of different dietary patterns on body weight loss and glucose improvement as previously reported [33, 34]. Briefly, in 2019, 253 participants were recruited in Shanghai, China, and the main inclusion criteria were aged 25–60 years, both sexes, BMI ≥ 24.0 kg/m^2^, and fasting plasma glucose ≥ 5.6 mmol/L. All ­participants received a 25% calorie-restricted diet providing 1600 kcal/day for men and 1300 kcal/day for women on 5 days per week for 6 months. For the present study, 235 participants who completed 6-month intervention with plasma samples were included, including 81 in the MD group, 81 in the TJD group, and 73 in the CD group. More detailed information for the three diets has been reported in our previous studies [33, 34].

### Anthropometric assessments

The body weight and height were measured by a single trained staff. BMI was calculated as body weight (kg) divided by the square of height (m). Information of lifestyle including smoking and drinking was collected through questionnaires. Total energy intake was calculated from weighed food records, and physical activity data were collected with a smartwatch.

### OGTT and evaluation of glucose metabolism

All participants underwent a 2-h 75 g OGTT after overnight fasting at baseline and at 3 and 6 months of the intervention. In the meanwhile, fasting blood samples and samples at 1 h and 2 h after consuming glucose were collected and kept at −80°C. Glucose and insulin were measured by using an automatic analyzer (Hitachi 7080) with commercial kits from Wako Pure Chemical Industries.

Insulin resistance was estimated by HOMA-IR using the following equation: HOMA-IR = fasting insulin (µIU/mL) × fasting glucose (mg/dL)/405. β-cell function was estimated by HOMA-β: HOMA-β = (360 × fasting insulin [μIU/mL])/(fasting glucose [mg/dL] – 63) [51].

### Lipidomic assessments

Plasma lipidomics at baseline, 3 months, and 6 months were measured by a high-throughput targeted liquid chromatography tandem mass spectrometry using electrospray ionization as mentioned in our previous study [34]. When triglycerides contain the same amounts of carbon atoms and double bonds but different fatty acyls, they were classified into separate lipid species. A total of 364 lipids were included in the final analysis after excluding missing values and discrete values, unless otherwise stated.

### Measurements of SPARC

At baseline and follow-ups, fasting plasma SPARC, 1-h and 2-h post-glucose loading plasma SPARC were assessed by using the ELISA kits (DY941-05, R&D Systems) [25]. In brief, plasma ­samples were diluted five times and measured according to the manufacturer’s protocols. Specifically, the same reference sample was included on each plate and measured in duplicate, serving as an internal standard across the experiments. Based on repeated assessments of this reference, the intra-assay coefficient of variation (CV) was 2.64% and the inter-assay CV was 2.09% [35]. All reported SPARC concentrations were normalized to this internal standard before statistical analysis, ensuring comparability across plates and minimizing technical variability. Missing values and discrete values were excluded from the analysis.

### Statistical analysis

Baseline characteristics are presented as mean ± SD for continuous variables and as *n* (%) for categorical variables. Differences among the three diet groups were assessed using χ^2^ tests for categorical variables and one-way ANOVA for continuous variables.

For repeated measurements, the repeated-measures analysis of variance (ANOVA) was applied to investigate the changes of plasma SPARC concentrations during the OGTT (0 h, 1 h, and 2 h) (Fig. 2) or intervention period (baseline, 3 months, and 6 months) (Fig. 3) in the three intervention groups, respectively, after adjustments for sex, baseline age, baseline BMI, total energy intake, physical activity, smoking, and drinking. To evaluate the improvement in cardiometabolic traits following treatment, the linear mixed-effects models (R package *lme4*, *lmer* function) were employed. HOMA-IR, HOMA-β, fasting insulin, and fasting glucose at different interventional time points (baseline, 3 months, and 6 months) were modeled as outcome variables; the diet group, time, and their interaction were included as fixed effects, with adjustment for sex, baseline age, baseline BMI, total energy intake, physical activity, smoking, and drinking (­Supplementary Table S1). Participant identifier (ID) was modeled as a random intercept to account for within-subject correlation. *Post hoc* pairwise comparisons were conducted using the R package *emmeans* with *Bonferroni* adjustment to control for multiple testing.

Generalized linear models (GLMs, *glm* function) were employed to examine the associations between baseline plasma SPARC levels and changes in HOMA-IR, HOMA-β, fasting insulin, fasting glucose, and lipid species from baseline to 3 or 6 months in the three groups, respectively. Model 1 was adjusted for sex, baseline age, baseline BMI, and the respective baseline value of each outcome. Model 2 was additionally adjusted for total energy intake, physical activity, smoking, and drinking. The associations are reported as standardized β ± SE after z-score normalization of the SPARC levels. To control for multiple comparisons, false discovery rate (FDR)–adjusted *P* values (*P*_FDR_) were calculated using the Benjamini–Hochberg method. Multiplicative interactions between baseline SPARC-1H and dietary group (SPARC-1H × group) were tested using GLMs. Causal mediation analyses (R package *mediation*, *mediate* function) were performed to assess the potential mediating effects of lipids. Missing data were handled according to the analysis type. For repeated-measures models, GLMs, and mediation analyses, a complete-case approach was used, excluding observations with missing values (*drop_na* function). For multiplicative interaction, mean imputation was applied for missing covariates to preserve statistical power.

All analyses were conducted in R (version 4.5.1), and two-sided *P* values < 0.05 were considered statistically significant.

## Supplementary Material

loaf039_Supplementary_Data

## Data Availability

The original data and datasets generated during the current study are not publicly available but are available from the corresponding author upon reasonable request.
