# Supplementary material for: Predictive value of 1-hour postprandial SPARC levels on metabolic outcomes from Mediterranean diet adherence: results from a randomized controlled feeding study
Source: Life Metab. 2025 Nov 13;5(2):loaf039. doi: 10.1093/lifemeta/loaf039 (PMC13124276; doi:10.1093/lifemeta/loaf039)
Supplement: loaf039_Supplementary_Data [file loaf039_supplementary_data.docx]

Supplementary Material for

**Predictive value of 1-hour postprandial SPARC levels on metabolic outcomes from Mediterranean diet adherence: results from a randomized controlled feeding study**

Yanru Chen^1,2^, Mengshan Ni^1,2^, Yufei Chen^1,2^, Chongrong Shen^1,2^, Yaogan Luo^3^, Huibin Lin^1,2^, Juan Zhang^1,2^, Huajie Dai^1,2^, Aibo Gao^1,2^, Muye Tong^1,2^, Yinmeng Zhu^1,2^, Yan Lu^4^, Jie Hong^1,2^, Weiqiong Gu^1,2^, Rong Zeng^5^, Weiqing Wang^1,2^, Xu Lin^3^, Min Xu^1,2^, Ruixin Liu^1,2^, Guang Ning^1,2^, Jiqiu Wang^1,2^

**Supplementary Table S1** Values of HOMA-IR, HOMA-Β, fasting insulin, and fasting glucose at baseline, 3 months, and 6 months.

|  |  | **MD** | **TJD** | **CD** | ***P*_group_** | ***P*_time_** | ***P*_group × time_** | |
| --- | --- | --- | --- | --- | --- | --- | --- | --- |
| HOMA-IR | Baseline | 4.13 ± 2.80 | 4.63 ± 3.47 | 4.40 ± 2.37 | 0.23 | 0.002 | | 0.57 |
|  | 3 months | 3.21 ± 2.16 | 3.86 ± 4.87 | 3.17 ± 1.42 |  |  |  |  |
|  | 6 months | 2.64 ± 1.29 | 3.36 ± 2.58 | 3.02 ± 1.66 |  |  |  |  |
| HOMA-β | Baseline | 109.49 ± 66.52 | 130.38 ± 79.03 | 117.98 ± 58.43 | 0.16 | 0.006 | | 0.57 |
|  | 3 months | 90.05 ± 52.63 | 108.77 ± 84.39 | 90.77 ± 35.87 |  |  |  |  |
|  | 6 months | 87.94 ± 41.46 | 111.48 ± 66.46 | 97.66 ± 48.57 |  |  |  |  |
| Fasting insulin, μIU/mL | Baseline | 14.72 ± 9.60 | 16.84 ± 11.67 | 15.69 ± 7.96 | 0.18 | 0.001 | | 0.50 |
|  | 3 months | 11.61 ± 6.81 | 14.01 ± 15.06 | 11.56 ± 4.74 |  |  |  |  |
|  | 6 months | 10.11 ± 4.77 | 12.83 ± 8.97 | 11.46 ± 5.97 |  |  |  |  |
| Fasting glucose, mmol/L | Baseline | 6.25 ± 0.73 | 6.10 ± 0.52 | 6.24 ± 0.80 | 0.47 | 0.0004 | | 0.83 |
|  | 3 months | 6.13 ± 0.58 | 6.01 ± 0.51 | 6.09 ± 0.61 |  |  |  |  |
|  | 6 months | 5.82 ± 0.41 | 5.77 ± 0.44 | 5.87 ± 0.5 |  |  |  |  |

Values of HOMA-IR, HOMA-β, fasting insulin, and fasting glucose at baseline, 3 months, and 6 months are presented as mean ± SD in the three groups. The effects of group, time, and their interaction were analyzed by linear mixed effect models, adjusting for sex, baseline age, baseline BMI, total energy intake, physical activity, smoking, and drinking.

**Supplementary Table S2** Plasma SPARC-1H levels at baseline and metabolic changes from baseline to 3 and 6 months in all participants.

|  |  | | | |  | | | **Baseline SPARC-1H** | | | | |  | |
| --- | --- | --- | --- | --- | --- | --- | --- | --- | --- | --- | --- | --- | --- | --- |
|  | | |  | **3 months** | | | | | | **6 months** | | |  | |
|  |  |  |  | **β** ± **SE** | | ***P* value** | | | ***P*_SPARC-1H × group_** | **β** ± **SE** | ***P* value** | | ***P*_SPARC-1H × group_** | |
| **Model 1** | | | | | | | | | | | | |  | |
| Metabolic changes | | HOMA-IR | | −0.03 ± 0.20 | | | 0.90 | | / | 0.25 ± 0.11 | | **0.026** | | **/** |
|  |  | HOMA-β | | −0.79 ± 3.36 | | | 0.82 | | / | 3.94 ± 2.72 | | 0.15 | | / |
|  |  | Fasting insulin, μIU/mL | | −0.05 ± 0.60 | | | 0.93 | | / | 0.78 ± 0.38 | | **0.040** | | **/** |
|  |  | Fasting glucose, mmol/L | | 0.02 ± 0.03 | | | 0.46 | | / | 0.04 ± 0.03 | | 0.12 | | / |
| **Model 2** | |  | |  | | |  | |  |  | |  | |  |
| Metabolic changes | | HOMA-IR | | −0.08 ± 0.21 | | | 0.72 | | / | 0.23 ± 0.12 | | 0.05 | | / |
|  |  | HOMA-β | | −0.78 ± 3.51 | | | 0.82 | | / | 3.62 ± 2.87 | | 0.21 | | / |
|  |  | Fasting insulin, μIU/mL | | −0.18 ± 0.63 | | | 0.78 | | / | 0.71 ± 0.40 | | 0.08 | | / |
|  |  | Fasting glucose, mmol/L | | 0.02 ± 0.03 | | | 0.58 | | / | 0.03 ± 0.03 | | 0.23 | | / |
| **Model 3** | |  | |  | | |  | |  |  | |  | |  |
| Metabolic changes | | HOMA-IR | | −0.08 ± 0.21 | | | 0.70 | | 0.96 | 0.26 ± 0.12 | | **0.027** | | **0.010** |
|  |  | HOMA-β | | −0.92 ± 3.55 | | | 0.80 | | 0.91 | 4.24 ± 2.91 | | 0.15 | | 0.33 |
|  |  | Fasting insulin, μIU/mL | | −0.19 ± 0.64 | | | 0.76 | | 0.98 | 0.81 ± 0.40 | | **0.043** | | **0.011** |
|  |  | Fasting glucose, mmol/L | | 0.01 ± 0.03 | | | 0.66 | | 0.71 | 0.04 ± 0.03 | | 0.15 | | 0.33 |

Data are presented as β ± SE after z-score normalization of baseline plasma SPARC-1H levels. The changes in HOMA-IR, HOMA-β, fasting insulin, and fasting glucose from baseline to 3 months and 6 months were calculated by the levels of clinical characteristics at 3 months and 6 months minus those at baseline. Baseline plasma SPARC-1H levels showed positive relationships with the changes of HOMA-IR and fasting insulin in 6-month follow-up both in Model 1 and Model 3. *P* values were calculated using generalized linear model (GLM).

Model 1: adjusted for sex, baseline age, baseline BMI, and respective baseline values for each outcome.

Model 2: based on Model 1 and further adjusted for total energy intake, physical activity, smoking, and drinking.

Model 3: based on Model 2 and further adjusted for diet groups.

**Supplementary Table S3** Fasting SPARC levels at baseline and metabolic changes from baseline to 3 months and 6 months in all participants.

|  |  | **Baseline fasting SPARC** | | | |
| --- | --- | --- | --- | --- | --- |
|  |  | **3 months** | | **6 months** | |
|  |  | **β** ± **SE** | ***P* value** | **β** ± **SE** | ***P* value** |
| **Model 1** |  |  |  |  |  |
| Metabolic changes | HOMA-IR | 0.06 ± 0.20 | 0.76 | 0.20 ± 0.11 | 0.08 |
|  | HOMA-β | −0.62 ± 3.38 | 0.85 | 1.13 ± 2.74 | 0.68 |
|  | Fasting insulin, μIU/mL | 0.16 ± 0.61 | 0.80 | 0.53 ± 0.38 | 0.17 |
|  | Fasting glucose, mmol/L | 0.04 ± 0.03 | 0.25 | 0.05 ± 0.03 | 0.07 |
| **Model 2** |  |  |  |  |  |
| Metabolic changes | HOMA-IR | 0.03 ± 0.22 | 0.90 | 0.15 ± 0.12 | 0.21 |
|  | HOMA-β | −1.09 ± 3.60 | 0.76 | 0.19 ± 2.97 | 0.95 |
|  | Fasting insulin, μIU/mL | 0.07 ± 0.65 | 0.91 | 0.39 ± 0.41 | 0.35 |
|  | Fasting glucose, mmol/L | 0.03 ± 0.03 | 0.30 | 0.04 ± 0.03 | 0.11 |
| **Model 3** |  |  |  |  |  |
| Metabolic changes | HOMA-IR | 0.03 ± 0.22 | 0.90 | 0.16 ± 0.12 | 0.18 |
|  | HOMA-β | −1.14 ± 3.62 | 0.75 | 0.46 ± 2.98 | 0.88 |
|  | Fasting insulin, μIU/mL | 0.07 ± 0.65 | 0.91 | 0.43 ± 0.41 | 0.30 |
|  | Fasting glucose, mmol/L | 0.03 ± 0.03 | 0.35 | 0.05 ± 0.03 | 0.09 |

Data are presented as β ± SE after z-score normalization of baseline fasting SPARC levels. There were no significant association between baseline fasting SPARC levels and the changes in HOMA-IR, HOMA-β, fasting insulin, and fasting glucose from baseline to 3 months or 6 months in all participants. *P* values were calculated using GLM.

Model 1: adjusted for sex, baseline age, baseline BMI, and respective baseline values for each outcome.

Model 2: based on Model 1 and further adjusted for total energy intake, physical activity, smoking, and drinking.

Model 3: based on Model 2 and further adjusted for diet groups.

**Supplementary Table S4** Plasma SPARC-2H levels at baseline and metabolic changes from baseline to 3 months and 6 months in all participants.

|  | |  | **Baseline SPARC-2H** | | | | |
| --- | --- | --- | --- | --- | --- | --- | --- |
|  | | | | **3 months** | | **6 months** | |
|  |  |  |  | **β** ± **SE** | ***P* value** | **β** ± **SE** | ***P* value** |
| **Model 1** |  | | |  |  |  |  |
| Metabolic changes | HOMA-IR | | | −0.09 ± 0.20 | 0.67 | 0.13 ± 0.14 | 0.35 |
|  | HOMA-β | | | −2.32 ± 3.41 | 0.50 | −0.44 ± 3.30 | 0.89 |
|  | Fasting insulin, μIU/mL | | | −0.28 ± 0.61 | 0.65 | 0.33 ± 0.46 | 0.48 |
|  | Fasting glucose, mmol/L | | | 0.03 ± 0.03 | 0.32 | 0.05 ± 0.03 | 0.15 |
| **Model 2** |  | | |  |  |  |  |
| Metabolic changes | HOMA-IR | | | −0.13 ± 0.21 | 0.55 | 0.13 ± 0.14 | 0.34 |
|  | HOMA-β | | | −2.26 ± 3.51 | 0.52 | −0.01 ± 3.47 | 0.99 |
|  | Fasting insulin, μIU/mL | | | −0.36 ± 0.63 | 0.57 | 0.35 ± 0.48 | 0.47 |
|  | Fasting glucose, mmol/L | | | 0.02 ± 0.03 | 0.50 | 0.05 ± 0.03 | 0.14 |
| **Model 3** |  | | |  |  |  |  |
| Metabolic changes | HOMA-IR | | | −0.13 ± 0.21 | 0.53 | 0.19 ± 0.14 | 0.19 |
|  | HOMA-β | | | −2.46 ± 3.52 | 0.49 | 0.97 ± 3.55 | 0.79 |
|  | Fasting insulin, μIU/mL | | | −0.38 ± 0.63 | 0.55 | 0.52 ± 0.49 | 0.29 |
|  | Fasting glucose, mmol/L | | | 0.02 ± 0.03 | 0.52 | 0.06 ± 0.03 | 0.08 |

Data are presented as β ± SE after z-score normalization of baseline SPARC-2H levels. There were no significant association between baseline SPARC-2H levels and the changes in HOMA-IR, HOMA-β, fasting insulin, and fasting glucose from baseline to 3 months or 6 months in all participants. *P* values were calculated using GLM.

Model 1: adjusted for sex, baseline age, baseline BMI, and respective baseline values for each outcome.

Model 2: based on Model 1 and further adjusted for total energy intake, physical activity, smoking, and drinking.

Model 3: based on Model 2 and further adjusted for diet groups.

**Supplementary Table S5** Fasting SPARC levels at baseline and metabolic changes from baseline to 3 months and 6 months in the MD, TJD and CD groups.

|  |  | **Baseline fasting SPARC** | | | | | | | |
| --- | --- | --- | --- | --- | --- | --- | --- | --- | --- |
|  |  | **MD** | | **TJD** | | | **CD** | | |
|  |  | **β ± SE** | ***P* value** | | **β ± SE** | ***P* value** | | **β ± SE** | ***P* value** |
| **Metabolic changes from baseline to 3 months** | | | | | | | | | |
| Model 1 | HOMA-IR | 0.20 ± 0.26 | 0.45 | | 0.32 ± 0.52 | 0.54 | | −0.11 ± 0.13 | 0.40 |
|  | HOMA-β | 1.63 ± 5.58 | 0.77 | | 2.55 ± 8.22 | 0.76 | | −2.81 ± 2.76 | 0.31 |
|  | Fasting insulin, μIU/mL | 0.64 ± 0.78 | 0.41 | | 0.89 ± 1.58 | 0.58 | | −0.36 ± 0.41 | 0.39 |
|  | Fasting glucose, mmol/L | 0.06 ± 0.07 | 0.38 | | 0.07 ± 0.05 | 0.20 | | −0.02 ± 0.05 | 0.72 |
| Model 2 | HOMA-IR | 0.14 ± 0.28 | 0.62 | | 0.48 ± 0.65 | 0.46 | | −0.07 ± 0.12 | 0.55 |
|  | HOMA-β | 1.79 ± 5.78 | 0.76 | | 4.75 ± 9.93 | 0.63 | | −2.95 ± 2.75 | 0.29 |
|  | Fasting insulin, μIU/mL | 0.52 ± 0.81 | 0.53 | | 1.43 ± 1.95 | 0.47 | | −0.27 ± 0.38 | 0.48 |
|  | Fasting glucose, mmol/L | 0.03 ± 0.07 | 0.63 | | 0.11 ± 0.06 | 0.08 | | 0.01 ± 0.05 | 0.92 |
| **Metabolic changes from baseline to 6 months** | | | | | | | | | |
| Model 1 | HOMA-IR | 0.22 ± 0.15 | 0.15 | | 0.75 ± 0.25 | **0.005** | | −0.08 ± 0.15 | 0.62 |
|  | HOMA-β | −1.03 ± 3.89 | 0.79 | | 11.39 ± 6.29 | 0.08 | | −3.06 ± 4.37 | 0.49 |
|  | Fasting insulin, μIU/mL | 0.58 ± 0.54 | 0.29 | | 2.30 ± 0.85 | **0.009** | | −0.31 ± 0.53 | 0.57 |
|  | Fasting glucose, mmol/L | 0.09 ± 0.05 | 0.07 | | 0.09 ± 0.05 | 0.07 | | −0.01 ± 0.04 | 0.82 |
| Model 2 | HOMA-IR | 0.19 ± 0.15 | 0.21 | | 0.75 ± 0.30 | **0.018** | | −0.08 ± 0.16 | 0.63 |
|  | HOMA-β | −1.42 ± 4.02 | 0.73 | | 13.73 ± 7.60 | 0.08 | | −2.73 ± 4.72 | 0.57 |
|  | Fasting insulin, μIU/mL | 0.49 ± 0.55 | 0.38 | | 2.40 ± 1.03 | **0.025** | | −0.33 ± 0.56 | 0.56 |
|  | Fasting glucose, mmol/L | 0.07 ± 0.05 | 0.13 | | 0.08 ± 0.06 | 0.18 | | 0.02 ± 0.04 | 0.72 |

Data are presented as β ± SE after z-score normalization of baseline fasting SPARC levels in respective dietary groups. Baseline fasting SPARC levels were positively associated with the changes in HOMA-IR and fasting insulin from baseline to 6 months in the TJD group. No other significant differences were observed. *P* values were calculated using GLM.

Model 1: adjusted for sex, baseline age, baseline BMI, and respective baseline values for each outcome.

Model 2: based on Model 1 and further adjusted for total energy intake, physical activity, smoking, and drinking.

**Supplementary Table S6** Plasma SPARC-2H levels at baseline and metabolic changes from baseline to 3 months and 6 months in the MD, TJD and CD groups.

|  |  | **Baseline SPARC-2H** | | | | | | |
| --- | --- | --- | --- | --- | --- | --- | --- | --- |
|  |  | **MD** | | **TJD** | | | **CD** | |
|  |  | **β** ± **SE** | ***P* value** | | **β** ± **SE** | ***P* value** | **β** ± **SE** | ***P* value** |
| **Metabolic changes from baseline to 3 months** | | | | | | | | |
| Model 1 | HOMA-IR | 0.25 ± 0.23 | 0.28 | | −0.05 ± 0.53 | 0.92 | −0.21 ± 0.14 | 0.14 |
|  | HOMA-β | 2.50 ± 5.20 | 0.63 | | 0.39 ± 8.25 | 0.96 | −5.25 ± 2.95 | 0.08 |
|  | Fasting insulin, μIU/mL | 0.81 ± 0.68 | 0.24 | | −0.09 ± 1.60 | 0.96 | −0.75 ± 0.43 | 0.09 |
|  | Fasting glucose, mmol/L | 0.04 ± 0.07 | 0.60 | | 0.02 ± 0.05 | 0.77 | 0.01 ± 0.06 | 0.85 |
| Model 2 | HOMA-IR | 0.26 ± 0.25 | 0.30 | | −0.10 ± 0.59 | 0.86 | −0.23 ± 0.13 | 0.08 |
|  | HOMA-β | 2.23 ± 5.39 | 0.68 | | 1.81 ± 8.82 | 0.84 | −5.15 ± 2.93 | 0.09 |
|  | Fasting insulin, μIU/mL | 0.83 ± 0.71 | 0.25 | | −0.15 ± 1.76 | 0.93 | −0.76 ± 0.40 | 0.06 |
|  | Fasting glucose, mmol/L | 0.04 ± 0.07 | 0.60 | | −0.001 ± 0.06 | 0.98 | −0.0003 ± 0.06 | 0.99 |
| **Metabolic changes from baseline to 6 months** | | | | | | | | |
| Model 1 | HOMA-IR | 0.22 ± 0.14 | 0.13 | | 0.33 ± 0.34 | 0.34 | −0.36 ± 0.31 | 0.26 |
|  | HOMA-β | 3.13 ± 3.69 | 0.40 | | 3.66 ± 8.13 | 0.66 | −14.31 ± 8.77 | 0.11 |
|  | Fasting insulin, μIU/mL | 0.71 ± 0.51 | 0.17 | | 1.00 ± 1.13 | 0.38 | −1.37 ± 1.10 | 0.22 |
|  | Fasting glucose, mmol/L | 0.04 ± 0.04 | 0.32 | | 0.05 ± 0.06 | 0.42 | 0.06 ± 0.08 | 0.47 |
| Model 2 | HOMA-IR | 0.23 ± 0.14 | 0.12 | | 0.48 ± 0.36 | 0.19 | −0.22 ± 0.36 | 0.54 |
|  | HOMA-β | 3.19 ± 3.77 | 0.40 | | 5.23 ± 8.90 | 0.56 | −10.22 ± 10.34 | 0.33 |
|  | Fasting insulin, μIU/mL | 0.73 ± 0.51 | 0.16 | | 1.42 ± 1.22 | 0.25 | −0.88 ± 1.29 | 0.50 |
|  | Fasting glucose, mmol/L | 0.04 ± 0.05 | 0.34 | | 0.07 ± 0.07 | 0.33 | 0.05 ± 0.09 | 0.60 |

Data are presented as β ± SE after z-score normalization of baseline SPARC-2H levels in respective dietary groups. No significant differences were observed in all three groups. *P* values were calculated using GLM.

Model 1: adjusted for sex, baseline age, baseline BMI, and respective baseline values for each outcome.

Model 2: based on Model 1 and further adjusted for total energy intake, physical activity, smoking, and drinking.

**Supplementary Table S7** Mediation analysis of lipids between the associations of baseline SPARC-1H levels and metabolic changes.

|  | **Direct effects** | ***P*_dire_** | **Mediating effects** | ***P*_med_** | **Proportion of**  **mediating effects** | ***P*_prop_** |
| --- | --- | --- | --- | --- | --- | --- |
| **Path: baseline SPARC-1H → changes in lipids from baseline to 3 months → changes in HOMA-IR from baseline to 6 months** | | | | | | |
| PE(P-18:0/16:0) | 0.007 | **0.012** | 0.001 | 0.34 | 7.3% | 0.34 |
| PE(P-18:0/20:3) | 0.006 | **0.022** | 0.003 | **0.006** | 31.2% | **0.012** |
| PE(P-18:1/20:3) | 0.006 | **0.034** | 0.003 | **0.036** | 29.4% | **0.040** |
| PE(P-16:0/22:5) | 0.006 | **0.014** | 0.002 | **0.014** | 25.1% | **0.016** |
| PE(P-18:1/20:4) | 0.008 | **0.006** | 0.001 | 0.15 | 11.7% | 0.15 |
| PE(O-16:0/20:4) | 0.008 | **0.002** | 0.001 | 0.15 | 10.0% | 0.15 |
| PE(O-18:0/20:3) | 0.007 | **0.010** | 0.001 | 0.24 | 14.2% | 0.25 |
| PE(O-16:0/22:5) | 0.007 | **0.006** | 0.001 | 0.09 | 14.8% | 0.09 |
| PE(O-18:0/22:4) | 0.007 | **0.006** | 0.001 | 0.27 | 8.3% | 0.28 |
| **Path: baseline SPARC-1H → changes in lipids from baseline to 3 months → changes in HOMA-β from baseline to 6 months** | | | | | | |
| PE(P-18:0/16:0) | 0.098 | 0.17 | 0.019 | 0.45 | 12.4% | 0.48 |
| PE(P-18:0/20:3) | 0.073 | 0.33 | 0.048 | 0.09 | 35.2% | 0.19 |
| PE(P-18:1/20:3) | 0.077 | 0.34 | 0.045 | 0.18 | 32.6% | 0.26 |
| PE(P-16:0/22:5) | 0.088 | 0.23 | 0.022 | 0.40 | 15.4% | 0.47 |
| PE(P-18:1/20:4) | 0.110 | 0.12 | 0.019 | 0.41 | 12.4% | 0.44 |
| PE(O-16:0/20:4) | 0.124 | 0.08 | 0.012 | 0.49 | 6.0% | 0.50 |
| PE(O-18:0/20:3) | 0.102 | 0.17 | 0.021 | 0.48 | 15.7% | 0.51 |
| PE(O-16:0/22:5) | 0.106 | 0.14 | 0.012 | 0.57 | 7.3% | 0.59 |
| PE(O-18:0/22:4) | 0.105 | 0.13 | 0.001 | 0.92 | 0.9% | 0.91 |
| **Path: baseline SPARC-1H → changes in lipids from baseline to 3 months → changes in fasting insulin from baseline to 6 months** | | | | | | |
| PE(P-18:0/16:0) | 0.022 | **0.012** | 0.004 | 0.26 | 13.3% | 0.27 |
| PE(P-18:0/20:3) | 0.017 | **0.042** | 0.009 | **0.016** | 33.7% | **0.022** |
| PE(P-18:1/20:3) | 0.018 | 0.06 | 0.009 | **0.036** | 31.0% | **0.040** |
| PE(P-16:0/22:5) | 0.019 | **0.032** | 0.007 | **0.030** | 26.6% | **0.032** |
| PE(P-18:1/20:4) | 0.025 | **0.006** | 0.004 | 0.20 | 12.4% | 0.20 |
| PE(O-16:0/20:4) | 0.026 | **0.002** | 0.004 | 0.17 | 10.9% | 0.17 |
| PE(O-18:0/20:3) | 0.022 | **0.016** | 0.005 | 0.27 | 16.0% | 0.28 |
| PE(O-16:0/22:5) | 0.021 | **0.020** | 0.004 | 0.12 | 15.4% | 0.13 |
| PE(O-18:0/22:4) | 0.023 | **0.014** | 0.003 | 0.31 | 8.5% | 0.31 |
| **Path: baseline SPARC-1H → changes in lipids from baseline to 3 months → changes in fasting glucose from baseline to 6 months** | | | | | | |
| PE(P-18:0/16:0) | 0.001 | 0.13 | <0.001 | 0.37 | 14.8% | 0.39 |
| PE(P-18:0/20:3) | 0.001 | 0.16 | <0.001 | 0.39 | 13.5% | 0.43 |
| PE(P-18:1/20:3) | 0.001 | 0.19 | <0.001 | 0.40 | 19.2% | 0.47 |
| PE(P-16:0/22:5) | 0.001 | 0.23 | <0.001 | 0.10 | 28.8% | 0.16 |
| PE(P-18:1/20:4) | 0.002 | 0.12 | <0.001 | 0.38 | 11.8% | 0.43 |
| PE(O-16:0/20:4) | 0.002 | 0.10 | <0.001 | 0.43 | 9.5% | 0.46 |
| PE(O-18:0/20:3) | 0.002 | 0.12 | <0.001 | 0.96 | 0.5% | 0.98 |
| PE(O-16:0/22:5) | 0.001 | 0.14 | <0.001 | 0.28 | 14.7% | 0.34 |
| PE(O-18:0/22:4) | 0.002 | 0.08 | <0.001 | 0.69 | 3.0% | 0.72 |

Mediation analyses were calculated with the changes of lipids from baseline to 3 months as mediators. Direct effects refer to the effects of path: baseline SPARC-1H to metabolic changes from baseline to 6 months. *P*_dire_ means *P* values for direct effects. *P*_med_ means *P* values for mediating effects. *P*_prop_ means *P* values for proportion of mediating effects. Analyses were adjusted by age, sex, baseline BMI, respective baseline values of lipids, total energy intake, physical activity, smoking, drinking, education, and waist.

**
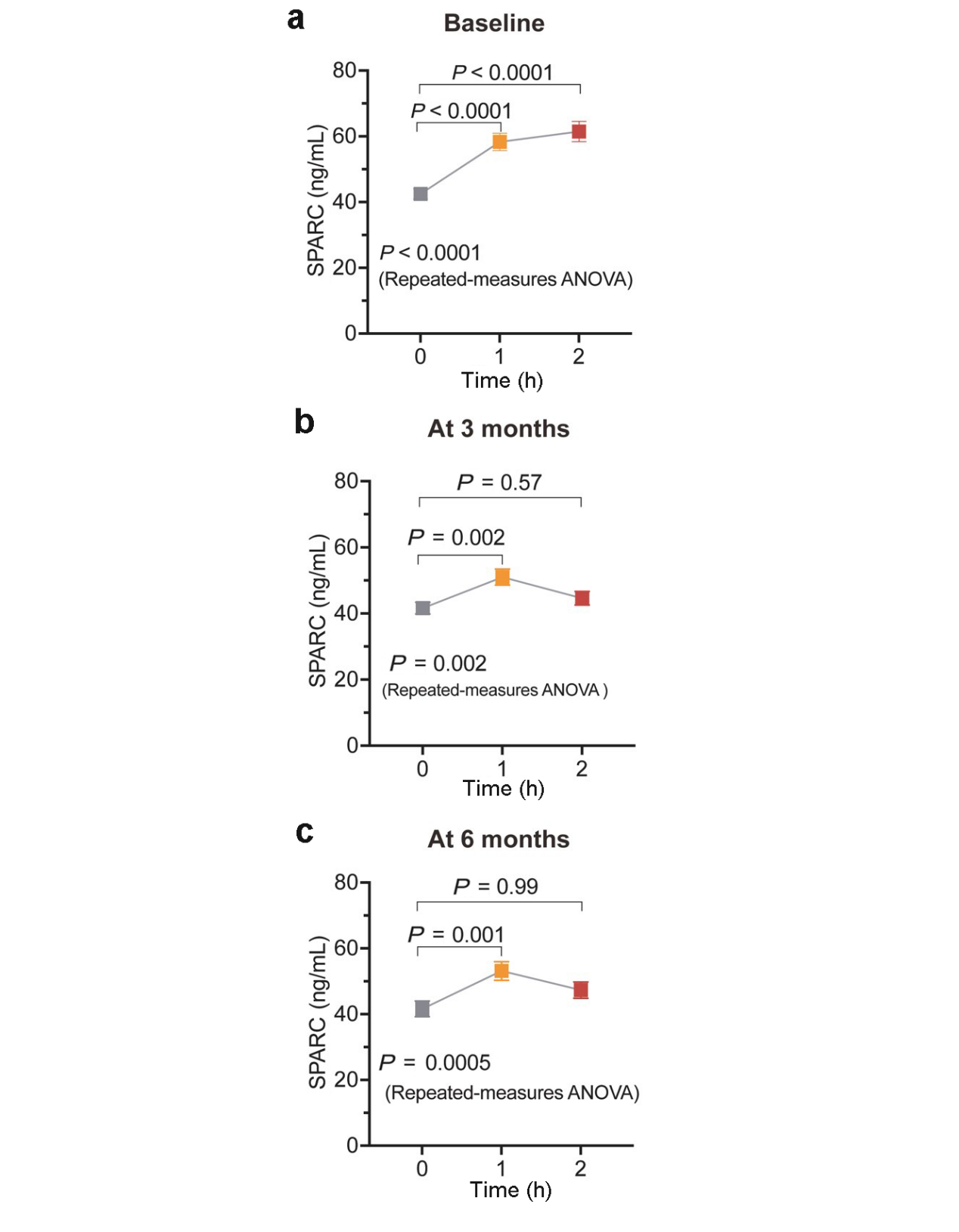
Supplementary Figure S1** Changes of SPARC levels during OGTT in all participants. (a**)** At baseline, plasma SPARC levels are increased greatly after oral glucose intake. (b and c) Plasma SPARC levels are increased significantly at OGTT-1H at 3 months (b) and 6 months (c). Comparations were calculated by repeated-measures ANOVA, adjusting for sex, baseline age, baseline BMI, total energy intake, physical activity, smoking, drinking, and dietary group. *Post hoc* pairwise comparisons between 0 h and 1 h or 2 h were conducted with Bonferroni adjustment.
